# Effect of 12-Week Vitamin D Supplementation on 25[OH]D Status and Performance in Athletes with a Spinal Cord Injury

**DOI:** 10.3390/nu8100586

**Published:** 2016-09-22

**Authors:** Joelle Leonie Flueck, Max Walter Schlaepfer, Claudio Perret

**Affiliations:** 1 Institute of Sports Medicine, Swiss Paraplegic Centre Nottwil, Nottwil 6207, Switzerland; claudio.perret@paraplegie.ch; 2 Institute of Human Movement Sciences and Sport, ETH Zurich, Zurich 8092, Switzerland; maxwschlaepfer@gmail.com

**Keywords:** 25[OH]D, spinal cord injuries, anaerobic performance test, dynamometer test

## Abstract

(1) Background: studies with able-bodied athletes showed that performance might possibly be influenced by vitamin D status. Vitamin D seems to have a direct impact on neuromuscular function by docking on vitamin D receptors in the muscle tissue. Additionally, a high prevalence of vitamin D deficiency was shown not only in infants and in the elderly but also in healthy adults and spinal cord injured individuals. Therefore, the aim of our study was to investigate whether a vitamin D dose of 6000 IU daily over 12 weeks would be sufficient to increase vitamin D status in indoor wheelchair athletes to a normal or optimal vitamin D level and whether vitamin D deficiency is associated with an impairment in muscle performance in these individuals; (2) Methods: vitamin D status was assessed in indoor elite wheelchair athletes in order to have a baseline measurement. If vitamin D status was below 75 nmol/L, athletes were supplemented with 6000 IU of vitamin D daily over 12 weeks. A vitamin D status over 75 nmol/L was supplemented with a placebo supplement. Vitamin D status, as well as a Wingate test and an isokinetic dynamometer test, were performed at baseline and after six and 12 weeks; (3) Results: 20 indoor elite wheelchair athletes participated in this double-blind study. All of these athletes showed an insufficient vitamin D status at baseline and were, therefore, supplemented with vitamin D. All athletes increased vitamin D status significantly over 12 weeks and reached an optimal level. Wingate performance was not significantly increased. Isokinetic dynamometer strength was significantly increased but only in the non-dominant arm in isometric and concentric elbow flexion; (4) Conclusion: a dose of 6000 IU of vitamin D daily over a duration of 12 weeks seems to be sufficient to increase vitamin D status to an optimal level in indoor wheelchair athletes. It remains unclear, whether upper body performance or muscle strength and vitamin D status are associated with each other.

## 1. Introduction

A high prevalence of vitamin D deficiency was shown not only in infants [1,2] and in the elderly [3], but also in young and healthy adults [4,5]. As vitamin D is primarily produced by ultraviolet radiation through sunlight exposure, a deficiency can possibly develop in healthy people as well. Such a deficiency may not only increase the risk for several different diseases, such as cancer [6,7], cardiovascular disease [8,9], and dementia [10], but also decrease neuromuscular function [11]. Such a neuromuscular impairment might be explained by the existence of vitamin D receptors (VDR) in human muscle tissue [12]. Thus, vitamin D deficiency might also lead to muscle weakness and pain. Further, vitamin D seems to influence not only muscle growth and cell differentiation, but also increase sarcoplasmic calcium uptake resulting in a higher muscle contractility [13]. Therefore, it is not surprising that positive effects of vitamin D supplementation on muscle function were found [14,15,16,17,18]. Studies showed a reduction in falls and a beneficial effects on muscle strength, balance, and gait performance in the elderly [19]. Other studies found an increase in upper and lower body muscle strength after vitamin D supplementation [18]. Nonetheless, the impact of vitamin D supplementation on muscular performance in athletes remains controversial. Some studies found no effect on performance [20,21], whereas others found a significantly increased isometric quadriceps strength, vertical jump, and sprint time after vitamin D supplementation [22,23].

Similar to the studies with able-bodied individuals, a high prevalence of vitamin D deficiency or insufficiency was found in patients [24] and athletes [25,26] with a spinal cord injury. Due to the impairment of the spinal cord, muscle strength might already be decreased and an additional impairment through vitamin D deficiency needs to be avoided. Only one study investigated the effect of vitamin D supplementation in athletes with a spinal cord injury on vitamin D status [27]. In this study, a vitamin D supplementation with 5000 IU daily increased vitamin D status over wintertime.

Therefore, the aim of our study was to investigate the effect of vitamin D supplementation on muscle strength and performance in indoor wheelchair athletes. Firstly, the objective was to detect whether a dose of 6000 IU daily is sufficient to increase vitamin D status to a normal level over 12 weeks in athletes suffering from a deficiency. Another goal was to investigate the relationship between vitamin D status and muscle strength.

## 2. Materials and Methods

### 2.1. Study Participants

Swiss male elite wheelchair indoor athletes, 18 to 60 years old and physically active for at least 45 min twice a week were recruited for this study. They had to perform their sport for more than two years and suffer from a chronic spinal cord injury or from cerebral palsy. The intervention study took place during the winter months (November–April) and the follow-up during spring (April–June) in Nottwil, Switzerland (47° north latitude). Any participant being abroad below the 37th parallel during the study phase or shortly before the start of the study was withdrawn from participating. Participants already supplementing with a vitamin D dose higher than 400 IU daily were also excluded from the study. Other exclusion criteria were suffering from a respiratory or cardiovascular disease, kidney insufficiency, or parathyroid gland ailment. All participants were asked to sign written informed consent and had to maintain their regular training schedule as well as to refrain from taking any additional supplements. The study was approved by the local ethics committee (Ethikkommission Nordwest-und Zentralschweiz (EKNZ), Basel, Switzerland) (Project #2015-344, clinicaltrials.gov NCT02621320).

### 2.2. Study Design

The double-blind, non-randomized intervention study took place at the Institute of Sports Medicine in Nottwil, Switzerland. Participants visited the institute on five different occasions during the intervention phase and on two additional occasions for those participating in the follow up. On the first visit, the screening questionnaire was completed and the medical history was checked to ensure that all criteria were fulfilled. The second visit was conducted in order to familiarize the participants with the performance tests. All participants performed and isokinetic dynamometer test (see Section 2.4) followed by a fifteen minute recovery break. Subsequently, a 30 s Wingate test on an arm crank ergometer (see Section 2.5) was performed.

Each participant replicated this test procedure on three occasions during the intervention phase and on two additional occasions during the follow up phase (only vitamin D concentration and the Wingate test). The tests took place at the same time of the day and were separated by six weeks. Before each session, the fulfillment of the test requirements was checked (i.e., no exercise twelve hours and no intense exercise 48 h before testing, at least seven hours of sleep during the previous night, no caffeine intake and replicated food intake prior to each session). After completion of this checklist, two venous blood samples were drawn in order to analyze the vitamin D and the calcium status. All participants with an insufficient vitamin D status (<75 nmol/L) received a vitamin D supplement during the intervention phase and all participants with a sufficient vitamin D status (>75 nmol/L) received a placebo supplement during the intervention phase (see Section 2.3). After the blood withdrawal, a Disabilities of the Arm, Shoulder and Hand (DASH) questionnaire [28] was completed.

### 2.3. Vitamin D Supplementation

Vitamin D3 (cholecalciferol) supplement (Vi-De 3^®^, Wild and Co. AG, Muttenz, Switzerland) was given in a dose of 6000 IU daily over twelve weeks (intervention phase). The tolerable upper limit intake level of the Endocrine Practice Guidelines Committee of 10,000 IU daily was not exceeded and, therefore, no side effects were expected [29]. The placebo supplement was based on the same alcohol solution (65% ethanol, Dr. Wild and Co. AG, Muttenz, Switzerland). The supplements were handed over in identical bottles and were ingested dropwise (either 60 drops or 1.3 mL daily). Bottles and solutions were not distinguishable for the participants in smell and color.

Self-reported compliance was assessed by regularly asking the frequency of taking vitamin D or placebo supplementation over the last two weeks. To achieve a high compliance, each participant installed a mobile app (Medisafe, Medisafe Inc., Boston, MA, USA) and set a daily reminder.

To assess tolerance, participants were asked every two weeks how they tolerate the supplement.

### 2.4. The Isokinetic Dynamometer Test

An isokinetic dynamometer (Cybex Norm II, Lumex Inc., Ronkomkoma, NY, USA) was used to measure peak torque of elbow flexion strength at different velocities for isometric (0°/s) and concentric (60°/s and 180°/s) exercise. The device was connected to the software (Humac 2015, CSMi, Stroughton, MA, USA) and calibration was performed in monthly intervals as proposed by the manufacturer. Participants were placed in a supine position fixed with straps and with a pillow under their knees. The shoulder joint was abducted in 45° and the wrist was strapped proximal with the hand in a neutral position to the lever arm of the dynamometer. The axis of rotation was aligned with the lateral epicondyle. Testing was limited between 20° and 120° of elbow flexion. Strong verbal encouragement was used during maximal effort.

A standardized warm-up with 10 repetitions at 120°/s was performed before the data collection. Subsequently, data collection started with the measurement of concentric work at 60 and 180°/s followed by isometric work.

Test-re-test reliability was checked prior to the start of the study in 10 able-bodied participants. Isometric, as well as both concentric measurements, showed “high” to “very high” reliability according to Munro’s classification of the intra-class correlation coefficient (ICC) [30]. The ICC ranged between 0.843 and 0.925 for the different test settings (Table S1).

### 2.5. The Wingate Test

Participants performed a Wingate test using a rotational speed-dependent arm crank ergometer (Angio V2, Lode B.V., Groningen, The Netherlands) which was connected to the software (Wingate, Lode B.V., Groningen, The Netherlands). This Wingate test on the arm crank ergometer was shown to be highly reliable in individuals with a paraplegia [31] and tetraplegia [32]. Participants were seated in an adapted office chair, which was positioned to allow a slight bend of the elbows. The height of the crank and the distance between the chair and the crank were recorded to replicate the conditions in the next test session. In some participants hand fixations and chest straps were needed to fix them to the crank or the chair. A resistance load of 1%–3% was applied in individuals with a tetraplegia [32]. In individuals with paraplegia, a resistance load of 4% was used. These settings were tested during the familiarization trials and adjusted for the intervention sessions where needed.

Five minutes of a standardized warm-up at 20 W and 60 rpm was performed before the start of the test. Subsequently, the resistance load was applied and the Wingate test was started. After the test, participants stopped to crank immediately and blood lactate concentrations were measured at 0, 2, 4, 6, 8, and 10 min after the end of the test using an enzymatic amperometric chip sensor system (Biosen C-Line Clinic, EKF diagnostic GmbH, Cardiff, UK). Blood samples were taken from the earlobe. A heart rate monitor (S610i, Polar Electro Oy, Kempele, Finland) was used to measure maximal heart rate during the test. These data were analyzed with the Polar Pro Trainer 5 software (Polar Electro Oy, Kempele, Finland). Rated perceived exertion (RPE) was assessed during warm-up and at the end of the test by using a Borg scale ranging from 6 to 20 [33]. Maximal power (P_peak_), average power (P_mean_) and fatigue index (FI) during the Wingate test were analyzed.

### 2.6. Blood Parameters

Blood samples were drawn from the antecubital vein using a blood collection system (S-Monovette^®^ 4.9 mL Z, Sarestedt, Nümbrecht, Germany). Samples were immediately packed into an opaque plastic tube to protect them from any ultraviolet radiation. The samples were then immediately centrifuged at 20 °C at 3000 rpm for 10 min (Rotina 380, Hettich GmbH, Tuttlingen, Germany) in the in-house laboratory of the Swiss Paraplegic Centre, Nottwil, Switzerland. After centrifugation, the samples were stored at −25 °C for later analysis.

Serum 25-hydroxyvitamin D (25[OH]D) was analyzed with an automated benchtop immunoanalyzer (Vidas^®^, bioMérieux, Marcy l’Etoile, France) using enzyme-linked fluorescent assay (ELFA). Serum calcium concentration was assayed with a photometric technique method (Cobas c501, Roche Diagnostic GmbH, Mannheim, Germany).

### 2.7. DASH Questionnaire

The Disabilities of the Arm, Shoulder and Hand (DASH) questionnaire was used to assess upper extremity function and symptoms (non-specific for wheelchair users) [28]. The DASH questionnaire was completed at the laboratory previously to the start of the Wingate test.

### 2.8. Data Analysis

Statistical analysis was performed using the software IBM SPSS Statistics Version 23.0 for Windows (IBM, Armonk, NY, USA). Statistical significance was set at an α-level of 0.05. Distribution of our data was tested by using the Kolmogorov-Smirnov, the Shapiro-Wilk test and the Q-Q plot. The results indicated, that all of your data was normally distributed except for the isokinetic dynamometer test and for the analysis of the follow up phase. For normally distributed data mean ± standard deviation (SD) was used. Not normally distributed data is presented as median [minimum; maximum]. To analyze differences in the mean of the outcome parameters between different time points, a one-way repeated-measurement ANOVA was performed for normally distributed data and the Brunner model [34] was applied for nonparametric data. Pairwise *t*-tests and Wilcoxon post hoc test were performed as post hoc analysis in normal and nonparametric data, respectively. In the case of multiple testing, Bonferroni corrections were applied. Tests six weeks after supplementation were called “intermediate” whereas tests after 12 weeks were called “post”. The first measurement during the follow up after six weeks is called “follow up 1” and the second follow up tests after 12-week placebo supplementation are called “follow up 2”. Spearman correlation was used to correlate the increase in vitamin D with the difference of peak elbow flexion at baseline and post. Pearson correlation was used to correlate the increase in vitamin D with the difference in peak power and mean power from baseline to post.

## 3. Results

Twenty-one healthy, male Swiss elite wheelchair indoor athletes participated in this study. Athletes were competing in wheelchair rugby (*n* = 15), basketball (*n* = 4), or table tennis (*n* = 2). One participant had to be excluded from data analysis due to non-compliancy. Therefore, twenty participants were included into data analysis. Ten out of these twenty participants agreed to take part in the follow up study (Table 1).

### 3.1. Vitamin D and Calcium Status

**Figure 1:**
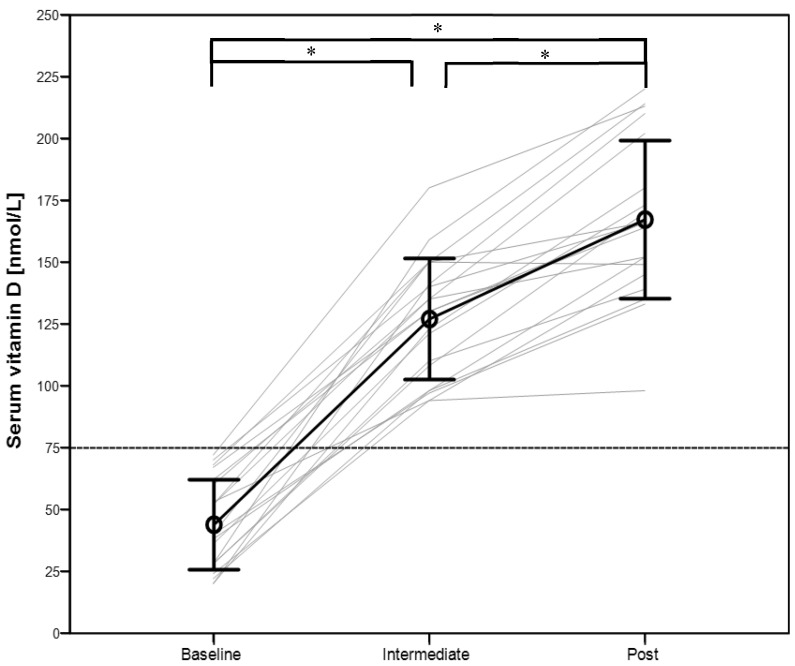
Serum vitamin D concentration at baseline, after six (intermediate) and 12 (post) weeks following vitamin D supplementation. * = significant difference (*p* < 0.05), data presented as mean and standard deviation, grey lines represent individual data.

**Figure 2:**
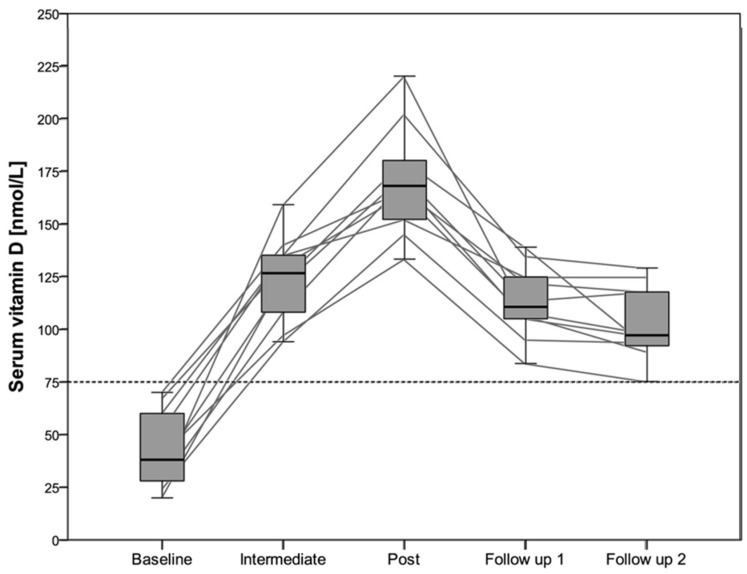
Serum vitamin D concentrations during the intervention and the follow up in 10 participants. Grey lines represents individuals’ data, data presented as median with interquartile range.

All participants enrolled into the study showed an insufficient or deficient vitamin D status at the baseline measurement (Figure 1). Therefore, no placebo group could be formed. Nineteen out of twenty athletes reached an optimal vitamin D status (100 to 220 nmol/L) after six weeks, and no one showed a toxic level (>375 nmol/L). Vitamin D status for the participants taking part in the follow up is shown in Figure 2. Significant differences were found between all different time points (*p* < 0.05). Calcium concentration was not significantly different between the three time points in the intervention study (*p* = 0.16) nor in the five time points, including the follow up data (*p* = 0.39). All calcium concentrations were within the normal physiological range (2.15 to 2.55 mmol/L).

### 3.2. Performance Tests

Significant improvements in the non-dominant arm were shown in isometric and 180°/s concentric exercise (Table 2).

**Figure 3:**
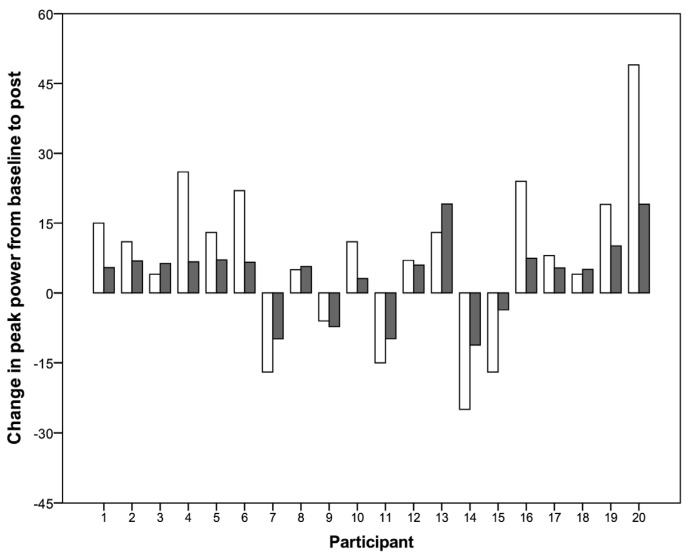
Individual changes in absolute (white bars in [W]) and relative (grey bars in [%]) of peak power from baseline to post measurement in the intervention study.

**Figure 4:**
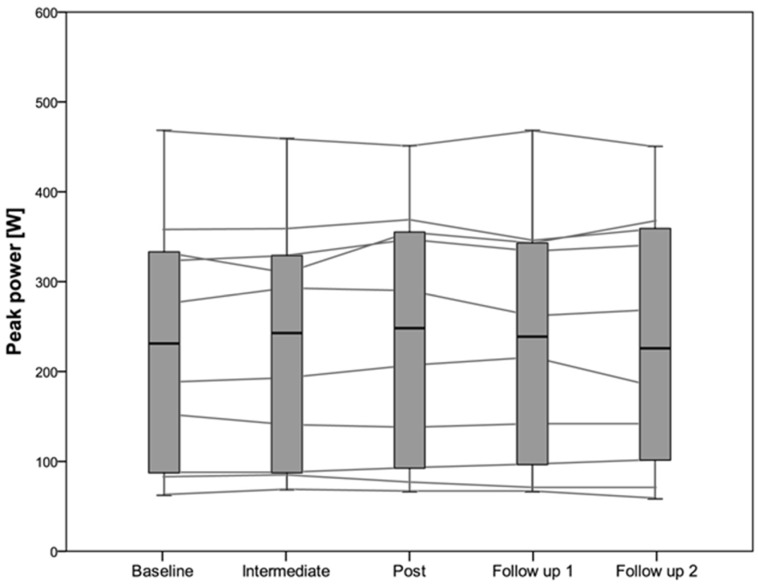
Peak power in 10 participants during the five measurements including the follow up study.

No significant differences in peak power were found over the three measurements during the intervention study (*p* = 0.09), nor during the follow-up study (*p* = 0.53). The same findings were shown for average power in the intervention (*p* = 0.13) and in the follow-up (*p* = 0.71) study. No significant differences were found in fatigue index (*p* = 0.15), maximal heart rate (*p* = 0.92), RPE (*p* = 0.76), and maximal lactate concentrations (*p* = 0.58) for the intervention study at the different time points (Table S2). Individual absolute and relative changes in peak power from baseline to post measurement in the intervention study are shown in Figure 3. The data for peak power in the follow up study is shown in Figure 4. Spearman correlation showed a significant correlation between the difference in the non-dominant arm at 60°/s and the increase of vitamin D from baseline to post (*p* = 0.01; *r_s_* = 0.564). This correlation coefficient (*r_s_*) reflects only “moderate” correlation. No other correlation for the dominant arm or the other exercise velocities showed any significant correlation. The increase in vitamin D status was significantly correlated with the difference in peak power from baseline to post (*p* = 0.044, *r* = 0.455). Again Pearson’s *r* reflects medium correlation. No significant correlation between the increase of vitamin D and mean power from baseline to post was found (*p* = 0.27; *r* = 0.258).

### 3.3. Other Outcome Parameters

No significant changes over time were found in the DASH score (*p* = 0.20) nor in the DASH sport score (*p* = 0.94) during the intervention phase. The participants reported a compliance of 97.3% over twelve weeks of vitamin D supplementation. Three out of twenty participants reported gastrointestinal side effects, such as a higher frequency of bowel movement and loose stool during vitamin D supplementation. Two out of the three showed the lowest compliance (75% and 82.1%) of all participants, and only one had a positive change in peak power from baseline to post measurement.

## 4. Discussion

A daily supplementation dosage of 6000 IU vitamin D seems to be sufficient to reach an optimal vitamin D status after 12 weeks in indoor athletes with an insufficient vitamin D status at baseline (Figure 1). After a 12 weeks follow up period with placebo supplementation, vitamin D status still was over 75 nmol/L but decreased significantly compared to the value at the end of the 12-week vitamin D supplementation period (Figure 2). The real effect of vitamin D supplementation on upper body exercise performance in athletes with a spinal cord injury still remains unclear due to a lack of a placebo group in our study.

### 4.1. Vitamin D Status

It is already well-known that a high amount of able-bodied and disabled Swiss athletes suffer from vitamin D deficiency or insufficiency during winter months [25,35]. The prevalence of vitamin D deficiency was even higher in indoor wheelchair athletes compared to outdoor athletes [25]. Nevertheless, it was surprising that all recruited athletes in the present study showed a deficient vitamin D status at baseline (44 ± 18 nmol/L). Oral vitamin D supplementation of 6000 IU daily over a 12-week time period was sufficient to increase vitamin D status to an optimal level (167 ± 24 nmol/L). The calculated slope of the relation between the vitamin D change and the supplementation was 2.05 nmol/L per 100 IU in the present study. This is slightly higher than the proposed slopes of 1.48 nmol/L per 100 IU [36] or 1.75 nmol/L per 100 IU [37]. The lower baseline vitamin D status in our study might explain this slight discrepancy.

Only a few other studies investigated the effect of vitamin D supplementation on vitamin D status in individuals with a spinal cord injury [27,38,39]. Most of these studies failed to achieve a sufficient vitamin D status after the supplementation period [39,40]. In one study [39], over 80% of the participants remained insufficient after vitamin D supplementation with 2000 IU daily over a two-week period. Similar findings were shown with a supplementation of 800 IU daily over a duration of one year [39], and in a study where the compliance for taking vitamin D supplements was only at 72% [40]. Our study showed not only a high compliance but also that a dosage of 6000 IU vitamin D daily over six weeks would be sufficient to increase vitamin D status to a normal level. Nevertheless, an individual approach needs to be applied, as a large individual variability occurred in the intermediate and post measurement of vitamin D status. Concerns whether such a high dose of vitamin D might be problematic were eliminated by the fact that calcium concentration remained in the normal range in our study. In addition, Heaney, Davies, Chen, Holick, and Barger-Lux [37] showed that a dose of 10,000 IU daily over five months was a safe intervention. Therefore, the Endocrine Society set their tolerable upper limit of 10,000 IU daily [29].

The dose of 6000 IU daily over 12 weeks seems to be sufficient and safe for its application in athletes with a spinal cord injury. Due to the fact that vitamin D status decreased during the 12-week follow up under placebo supplementation, we recommend to re-evaluate vitamin D status after a certain time period (e.g., 18 week after the end of the supplementation period).

### 4.2. Vitamin D and Muscle Performance

The present study did not find any significant increase in anaerobic Wingate test performance, although vitamin D status significantly increased over these 12 weeks. Fifteen out of twenty athletes showed an increase in peak power over this period (Figure 3). A significant increase in isokinetic strength in the non-dominant arm was found comparing baseline and post supplementation measurement (Table 2). Even though this increase was significant, it is not obvious, whether it was due to a training adaptation or due to the increased vitamin D status. The lack of a placebo or control group prevents drawing further conclusions. Similar results were shown by Pritchett, Pritchett, Ogan, Bishop, Broad, and LaCroix [26], whereas vitamin D status did not correlate with 20 m sprint or handgrip strength in wheelchair athletes. A recent meta-analysis [18] found a significant increase in upper limb strength in able-bodied participants favoring the supplementation compared to the placebo intervention. Two of these studies applied a relatively high dose (60,000 and 14,000 IU vitamin D per week) over four to six months [23,41]. A single bolus of 150,000 IU vitamin D increased quadriceps muscle strength of elite judokas significantly after eight days [42]. In contrast, a dose of 2000 IU vitamin D daily over 12 weeks did not significantly increase swimming performance as well as arm-grip strength and one-legged balance in adolescent swimmers [20]. Thus, no conclusive results were found for upper body muscle strength after vitamin D supplementation.

Similar to our results, Hamilton, et al. [43] found a significantly increased peak torque in the non-dominant leg in professional soccer players, but no increase in the dominant leg. Overall, no consistent association between vitamin D and isokinetic strength was found in this study. It remains unclear why, in the non-dominant leg in this study, and the non-dominant arm in our study, muscle strength improved in contrast to the dominant one. Further studies are needed to elucidate this issue as well as to investigate the effects of vitamin D on upper body muscle strength and handgrip function.

In addition, it is known that vitamin D binds to vitamin D receptors in the muscle tissue in order to turn up gene expression of type II muscle fibers [13]. A study performed with an elderly vitamin D deficient population showed a decreased atrophy of type II muscle fibers under vitamin D supplementation [44]. The relative number and size of type II muscle fibers was increased and muscle strength was improved. In addition, a reduction of falls and hip fractures occurred in the intervention group. This study showed, that the type II muscle fibers are possibly more affected compared to type I fibers. Knowing that, in individuals with a spinal cord injury type I muscle fibers are more predominant in the upper body [45], a smaller impact of vitamin D supplementation on these muscles might be expected. Again, such a speculation has to be further investigated in the future by means of muscle biopsies in order to determine the change in muscle fiber size and number after vitamin D supplementation.

### 4.3. Other Parameters

Our study did not find any significant change in the DASH score outcome over the supplementation period. This result suggests that the upper body impairment did not change over time and no additional injury occurred. Of course, due to the lack of a placebo group, this finding cannot be compared. A recent systematic review performed in healthy adults [46] revealed a small, but positive, effect on reducing the incidence of injuries when supplemented with vitamin D. Wyon, Koutedakis, Wolman, Nevill, and Allen [23] found a similar result in elite ballet dancers, who sustained significantly fewer injuries compared to the placebo group. Much more data is needed to finally conclude the impact of vitamin D supplementation on injury rate and severity of the injury. It is yet not clear whether there exists a positive association in general.

### 4.4. Limitations

The aim was to conduct a placebo-controlled intervention study. Unfortunately, all recruited participants showed an insufficient vitamin D status and were enrolled into the vitamin D supplementation group. The number of participants was limited due to the lack of further elite wheelchair athletes and due to the stringent inclusion or exclusion criteria. Even though an a priori power analysis showed a high power with 10 participants, this study might have been slightly underpowered due to a high variability in muscle force in athletes with a paraplegia or a tetraplegia. Therefore, it seems very difficult to draw final conclusions on how an insufficient vitamin D status impairs upper body performance or how a vitamin D supplementation might help to improve neuromuscular function. Nevertheless, this study showed clearly how vitamin D status increased over 12 weeks after supplementation and to which extent it decreased during the follow up. Further research is needed to investigate the relationship of vitamin D deficiency and neuromuscular performance in athletes with a spinal cord injury.

## 5. Conclusions

The present finding show, that a dose of 6000 IU vitamin D daily over 12 weeks is safe and sufficient to reach an optimal vitamin D level in indoor wheelchair athletes. Due to the lack of a placebo group, no final conclusion can be drawn whether vitamin D influences neuromuscular performance in athletes with a spinal cord injury. Isokinetic strength seems to have improved in the non-dominant arm over 12 weeks, but this finding might also result from continuous training.

## Figures and Tables

**Table 1:** Participants’ characteristics.

Participant	Training (h/Week)	Age (Years)	Height (cm)	Weight (kg)	Lesion Level	AIS	Sport	Classification	Follow up
1	3.5	37	185	103	C7	D	WR	2.0	Yes
2	11.0	20	170	54	C6	A	WR	1.5	No
3	4.0	24	179	58	C6	B	WR	0.5	Yes
4	3.5	35	187	97	T4	A	WB	1.0	No
5	4.0	47	185	63	T1	A	WR	2.5	No
6	2.5	27	180	70	T4	A	WB	1.0	Yes
7	3.5	27	181	61	C6	B	WR	2.5	No
8	5.0	48	186	67	C6	B	WR	1.0	Yes
9	5.0	44	176	80	C6	A	WR	0.5	Yes
10	12.0	35	193	92	T1	C	WB	2.5	Yes
11	8.0	38	172	70	C7	D	WR	2.0	Yes
12	4.0	26	188	90	C6	A	WR	0.5	No
13	5.0	30	182	65	C6	B	WR	0.5	No
14	8.0	34	180	85	C5	C	WR	1.5	No
15	6.5	50	185	83	L3	A	WB	3.0	Yes
16	13.5	21	184	63	C7	D	WR	2.5	Yes
17	3.5	65	175	65	C6	C	WR	1.5	No
18	15.0	33	180	60	C6	A	PT	class 1	No
19	4.0	26	155	52	CP	-	WR	1.5	Yes
20	5.0	57	170	72	T5	D	PT	class 4	No
Mean ± SD	6.3 ± 3.7	36 ± 12	180 ± 8	72 ± 15	-	-	-	-	-

AIS = American Spinal Injury Association Impairment Scale; T = thoracic; L, lumbar; C = cervical, CP = cerebral palsy, WB = wheelchair basketball, WR = wheelchair rugby, PT = para table tennis.

**Table 2:** Peak elbow flexion [Nm] reached during isokinetic dynamometer measurements.

Mode	Arm	Baseline	Intermediate	Post	*p*-Value
isometric	dominant	65 [46; 96]	71 [46; 98]	72 [50; 100]	0.071
	non-dominant	64 [49; 104]	68 [46; 106] *	71 [49; 106] *	0.019
concentric 60°/s	dominant	46 [30; 77]	47 [35; 71]	47 [37; 73]	0.197
	non-dominant	47 [30; 73]	50 [33; 71]	49 [37; 75]	0.078
concentric 180°/s	dominant	34 [24; 61]	31 [24; 58]	33 [27; 61]	0.269
	non-dominant	34 [24; 53]	37 [24; 54] **	35 [26; 53] **	0.001

Data presented as median [minimum; maximum] from 20 participants. Significant differences compared to baseline measurement * *p* < 0.05 and ** *p* < 0.01.

## Supplementary Materials

The following are available online at http://www.mdpi.com/2072-6643/8/10/586/s1, Table S1: Test-retest reliability of the isokinetic dynamometer test in 10 able-bodied participants, Table S2: Parameters measured during the Wingate test at baseline, intermediate and post supplementation.
