# Supplementary material for: Effect of 12-Week Vitamin D Supplementation on 25[OH]D Status and Performance in Athletes with a Spinal Cord Injury
Source: Nutrients. 2016 Sep 22;8(10):586. doi: 10.3390/nu8100586 (PMC5083975; doi:10.3390/nu8100586)
Supplement: Supplementary file 1 [file nutrients-08-00586-s001.docx]

Supplementary Materials: Effect of 12-Week Vitamin D Supplementation on 25[OH]D Status and Performance in Athletes with a Spinal Cord Injury

Joelle Leonie Flueck, Max Walter Schläpfer and Claudio Perret

**Table S1.** Test-retest reliability of the isokinetic dynamometer test in 10 able-bodied participants.

| **Mode** | **Arm** | **ICC** | **95% CI** | **SRD %** | **CV %** |
| --- | --- | --- | --- | --- | --- |
| concentric 60°/s | dominant | 0.922 | [0.718; 0.980] | 4.0 | 3.59 |
|  | non-dominant | 0.925 | [0.729; 0.981] | 4.2 | 2.90 |
| concentric 180°/s | dominant | 0.839 | [0.480; 0.958] | 9.2 | 5.29 |
|  | non-dominant | 0.867 | [0.555; 0.965] | 7.7 | 3.83 |
| isometric | dominant | 0.843 | [0.489; 0.959] | 7.6 | 3.39 |
|  | non-dominant | 0.892 | [0.626; 0.972] | 5.3 | 3.10 |

**Table S2.** Parameters measured during the Wingate test at baseline, intermediate and post supplementation.

| **Parameter** | **Baseline** | **Intermediate** | **Post** | ***p*-Value** |
| --- | --- | --- | --- | --- |
| P_max_ [W] | 206 ± 118 | 210 ± 123 | 214 ± 123 | 0.090 |
| P_mean_ [W] | 149 ± 84 | 151 ± 86 | 152 ± 86 | 0.133 |
| FI [W/s] | 3.8 ± 2.5 | 3.9 ± 2.6 | 4.1 ± 2.6 | 0.147 |
| HR_max_ [min^−1^] | 137 ± 19 | 138 ± 20 | 137 ± 23 | 0.920 |
| RPE_max_ [1] | 18 ± 1 | 18 ± 1 | 18 ± 1 | 0.760 |
| L_max_ [mmol/L] | 6.06 ± 2.21 | 5.95 ± 2.18 | 6.12 ± 2.16 | 0.582 |

P_max_ = peak power, P_mean_ = average power, FI = fatigue index, HR = heart rate, RPE = rated perceived exertion, L = lactate concentration.
